# Maternal malaria but not schistosomiasis is associated with a higher risk of febrile infection in infant during the first 3 months of life: A mother-child cohort in Benin

**DOI:** 10.1371/journal.pone.0222864

**Published:** 2019-09-19

**Authors:** Gino Agbota, Katja Polman, Frank T. Wieringa, Maiza Campos-Ponce, Manfred Accrombessi, Emmanuel Yovo, Clémentine Roucher, Sem Ezinmègnon, Javier Yugueros Marcos, Laurence Vachot, Pierre Tissières, Achille Massougbodji, Nadine Fievet, Michel Cot, Valérie Briand

**Affiliations:** 1 MERIT, IRD, Université Paris 5, Sorbonne Paris Cité, Paris, France; 2 Centre d’Etude et de Recherche sur le Paludisme Associé à la Grossesse et à l’Enfance (CERPAGE), Cotonou, Bénin; 3 Department of Biomedical Sciences, Institute of Tropical Medicine, Antwerp, Belgium; 4 Section Infectious Diseases, Department of Health Sciences, VU Amsterdam, Amsterdam, The Netherlands; 5 Nutripass, UMR204, Institut de Recherche pour le Développement, IRD/UM/SupAgro, Montpellier, France; 6 Medical Diagnostic Discovery Department (MD3), bioMérieux, Marcy l’Etoile, France; 7 UMR 9198, Institut de biologie Intégrative de la Cellule, Université Paris Saclay, Paris, France; Federal University of Agriculture, Abeokuta, NIGERIA

## Abstract

**Background:**

Malaria and schistosomiasis represent two of the most prevalent and disabling parasitic infections in developing countries. Few studies have evaluated the effect of maternal schistosomiasis and malaria in the peri-conceptional period on infant’s risk of infection.

**Methods:**

In Benin, women were followed from the preconception period until delivery. Subsequently, their children were followed from birth to 3 months of age. Pre-pregnancy malaria, malaria in pregnancy (MiP)—determined monthly using a thick blood smear—and urinary schistosomiasis—determined once before pregnancy and once at delivery using urine filtration—were the main maternal exposures. Infant’s febrile infection (fever with respiratory, gastrointestinal and/or cutaneous clinical signs anytime during follow-up) was the main outcome. In a secondary analysis, we checked the relation of malaria and schistosomiasis with infant’s hemoglobin (Hb) concentration. Both effects were separately assessed using logistic/mixed linear regression models.

**Results:**

The prevalence of MiP was 35.7% with 10.8% occurring during the 1^st^ trimester, and the prevalence of schistosomiasis was 21.8%. From birth to 3 months, 25.3% of infants had at least one episode of febrile infection. In multivariate analysis, MiP, particularly malaria in the 1^st^ trimester, was significantly associated with a higher risk of infant’s febrile infection (aOR = 4.99 [1.1; 22.6], p = 0.03). In secondary results, pre-pregnancy malaria and schistosomiasis were significantly associated with a lower infant’s Hb concentration during the first 3 months.

**Conclusion:**

We evidenced the deleterious effect of maternal parasitic infections on infant’s health. Our results argue in favor of the implementation of preventive strategies as early as in the peri-conception.

## Introduction

According to the Developmental Origins of Health and Diseases (DOHaD) concept, a deleterious environment during peri-conception, gestation and the early postnatal period could lead to a predisposition to childhood and adult-onset diseases [1]. Maternal infection and malnutrition during pregnancy are recognized stress factors that impact on the child health in high-income countries [2]. While infections as well as malnutrition in mid-pregnancy have been associated with fetal growth impairment, recent evidence suggests that infections and malnutrition before conception or early in pregnancy may also be deleterious for the fetus [3–5].

Malaria remains a major infectious disease for population in low and middle-income countries (LMICs). In 2017, 210 million cases of malaria were estimated, mainly occurring in sub-Saharan Africa (SSA) (92%) [6]. Malaria in pregnancy (MiP) is frequent and has already been associated with poor maternal and child outcomes. A recent review of studies conducted in SSA reported an overall prevalence of MiP of 38.2% (95% CI: 32.3%-44.1%) [7]. In addition to its well-known effects on maternal anemia and low birthweight, MiP has been associated with an enhanced susceptibility to malaria as well as to other infections in infancy [8–10]. In these studies, MiP mainly referred to malaria at delivery, none of them assessed the effect of malaria before conception or in early pregnancy on infant’s health.

On another note, schistosomiasis represents one of the most prevalent and disabling parasitic infections in LMICs. According to the World Health Organization (WHO), schistosomiasis affects more than 200 million people in LMICs in 2016 [11]. The prevalence and effects of schistosomiasis during pregnancy have been far less documented. In particular, schistosomiasis has been associated with placental inflammation, which could result in poor birth outcomes [12].

We aimed to assess the effect of maternal urinary schistosomiasis and malaria in the peri-conceptional period on the risk of febrile infection in infant during the first 3 months of life. In a secondary analysis, we also checked the effect of these two conditions on infant’s hemoglobin (Hb) concentration.

## Methods

### Study site, population and procedures

Between June 2014 and August 2018, a preconceptional mother-child cohort was conducted in Southern Benin in the districts of Sô-Ava and Akassato, 25 km from Cotonou. Sô-Ava is a lakeside area where fishing is the main activity of the population, while Akassato is a semi-rural area. Both areas are hyperendemic for malaria and schistosomiasis [13]. This study was carried out within the framework of two projects, RECIPAL (“REtard de Croissance Intra-utérin et PALudisme”, study protocol described elsewhere [14]) and SEPSIS (“Neonatal immune function and risk of sepsis in infants in a malaria endemic area”). Women of reproductive age were recruited and followed monthly until becoming pregnant; pregnant women were then followed throughout the pregnancy (RECIPAL). Then, a subset of infants born from the RECIPAL mothers was followed during the first 3 months of age (SEPSIS).

#### Preconception period

At enrolment, demographic, socioeconomic, anthropometric (weight, height, mid-upper-arm circumference) data and household characteristics were collected. Women were screened for urinary schistosomiasis and malaria. All women who were positive for schistosomiasis and malaria were immediately treated. Hemoglobin (Hb), c-reactive protein (CRP) and alpha-1-acid glycoprotein (AGP) levels were determined. Each month, women were visited at home, the first day of the last menstrual period was recorded and a urinary pregnancy test was performed.

#### Gestational follow up

Once pregnant, women were followed up monthly at the maternity clinic. At each antenatal care visit, anthropometric data, clinical data (temperature, blood pressure, intake of intermittent preventive treatment for malaria, use of insecticide treated net) and obstetrical data were collected. Women were screened monthly for malaria as well as for proteinuria and glycosuria. Maternal Hb, CRP and AGP levels were determined in the first and the third trimester of pregnancy. Urinary schistosomiasis was determined at delivery, and all women who were positive were treated after delivery. Malaria was also screened (using thick blood smear) at delivery in placental blood. In addition to the scheduled visits, women were invited to attend the maternity clinic anytime in case of symptoms.

#### Infant follow-up

A subset of infants (born from April 2016) was followed monthly from birth to 3 months of age. At birth and at each scheduled visit, anthropometric data (weight, length, head circumference and mid-upper-arm circumference), breastfeeding and dietary practices, vaccine coverage and clinical data (temperature, heart and respiratory rate and symptoms) were collected. Infants’ Hb concentration and malaria status (using a thick blood smear) were determined each month from birth until 3 months of age (4 time-points in total). During follow-up, mothers were encouraged to attend the health facility in case of any symptoms in the child. Clinical symptoms were recorded by the study’s nurses and then confirmed by the study’s referent physician. No biological investigation was carried out for etiological purposes of clinical symptoms.

#### Laboratory methods

Microscopic malaria was diagnosed by thick blood smear (TBS), and the parasitaemia was quantified using the Lambaréné method [15]. Diagnosis of urinary schistosomiasis was based on the microscopic detection of *S*. *haematobium* eggs in urine, using the filtration method [16]. Hb concentration was determined with a HemoCue^®^. CRP and AGP levels were determined by enzyme-linked immunosorbent assay (ELISA) technique [17]

### Definitions

#### Women

Pre-pregnancy malaria was defined as a positive TBS before pregnancy (yes/no). MiP was defined as at least one positive TBS during pregnancy (yes/no). In addition, we categorized malaria according to its timing during pregnancy as early-MiP (malaria in the 1^st^ trimester and not later on), late-MiP (malaria in the 2^nd^ or 3^rd^ trimester, but not in the 1^st^ trimester) and combined-MiP (malaria in the 1^st^ trimester combined with malaria in the 2^nd^ and/or 3^rd^ trimester). Schistosomiasis was defined as at least one *S*. *haematobium* eggs-positive urine sample before or during pregnancy (yes/no). Malaria-schistosomiasis coinfection was defined as the detection of both malaria and schistosomiasis at least once before or during pregnancy. Anemia during pregnancy was defined as an Hb level less than 11g/dL at least once during pregnancy (yes/no). Socioeconomic level was approximated using a score combining occupation and ownership of assets, which was then categorized according to the tertiles into low, medium and high categories. Gravidity was categorized as primi / secondigravidae (1–2) or multigravidae (≥3). Ethnicity was categorized as Toffin (main ethnic group) or others (including Aïzo, Fon and Yoruba ethnic groups). Educational level was categorized as literate if ≥ primary school or illiterate if not. Body mass index (BMI) was categorized as underweight (< 18.5 kg/m^2^), normal (18.5–24.9 kg/m^2^), overweight (25.0–29.9 kg/m^2^) and obesity (≥ 30 kg/m^2^) according to WHO standards. Gestational age estimation was based either on last menstrual period (LMP) if the difference between LMP and ultrasound scan (US) performed between 9 and 13 weeks of gestation was less than 7 days or on US if the difference was >7 days. At delivery, placental malaria was defined as a positive TBS of placental blood.

#### Infants

A febrile infection was defined as the combination of fever (rectal temperature ≥ 37.5°C) with respiratory (cough, dyspnea, rhinitis, bronchitis or abnormalities on auscultation), gastrointestinal (vomiting, diarrhea or abdominal pain), or cutaneous (skin fungi or skin rashes) clinical signs, or as clinical malaria (fever + positive thick blood smear). Infants were classified as whether they had at least one febrile infection during follow-up (yes/no). Hb concentration was considered as a quantitative and time-dependent variable from birth to 3 months of age. Feeding mode was categorized according to WHO standards into maternal breastfeeding (including both exclusive and predominant breastfeeding, the latter corresponding to water or water-based drinks consumed in addition to breast milk), mixed feeding and exclusive formula feeding [18]. Nutritional status of infants was assessed by weight-for-age, length-for-age and weight-for-length z-scores according to WHO standards, using macro for STATA [19].

### Statistical analysis

First, we described the general characteristics of the mother-child pairs according to maternal malaria and schistosomiasis.

Second, we used a logistic regression model to assess the effect of maternal schistosomiasis and malaria before and during pregnancy on the risk of infant’s febrile infection during the first 3 months of life.

The main exposure variables were maternal schistosomiasis, pre-pregnancy malaria and MiP. The adjustment variables were maternal age, gravidity, ethnicity, socioeconomic and educational level, anemia (before and during pregnancy) as well as infant’s sex, term at birth, breastfeeding, nutritional status and study center. In addition, sensitivity analyses were conducted to assess the effect of malaria-schistosomiasis coinfection (as the main exposure) on infant’s Hb concentration and risk of febrile infection.

In a secondary analysis, we assessed the effect of maternal schistosomiasis and malaria before and during pregnancy on the infant’s Hb concentration during the first 3 months of life using a mixed linear regression model with a random intercept at the individual level (considering that successive Hb concentrations in the same infant were correlated).

All variables with a p-value below 0.2 in univariate analysis were included in the multivariate analysis. Then, the variables with a p-value less than 0.05 after a step-by-step backward selection procedure were kept in the multivariate model. Malaria and schistosomiasis were forced in all final models. Statistical analyses were done with Stata version 13.1 for Windows (Stata Corp., College Station, TX).

### Ethics statement

The Ministry of Health in Benin and the Ethics Committee of the Institut des Sciences Biomédicales Appliquées in Benin approved RECIPAL (decision no. 39 of 05/16/ 2014) and SEPSIS (decision no. 85 of 04/05/2016) projects. Women were included in RECIPAL after providing a signed written informed consent, and the newborns were included in SEPSIS after both parents have provided a signed written informed consent. All infections detected (malaria and schistosomiasis) were immediately treated and all medications were paid by the projects.

## Results

### Study profile

**Fig 1:**
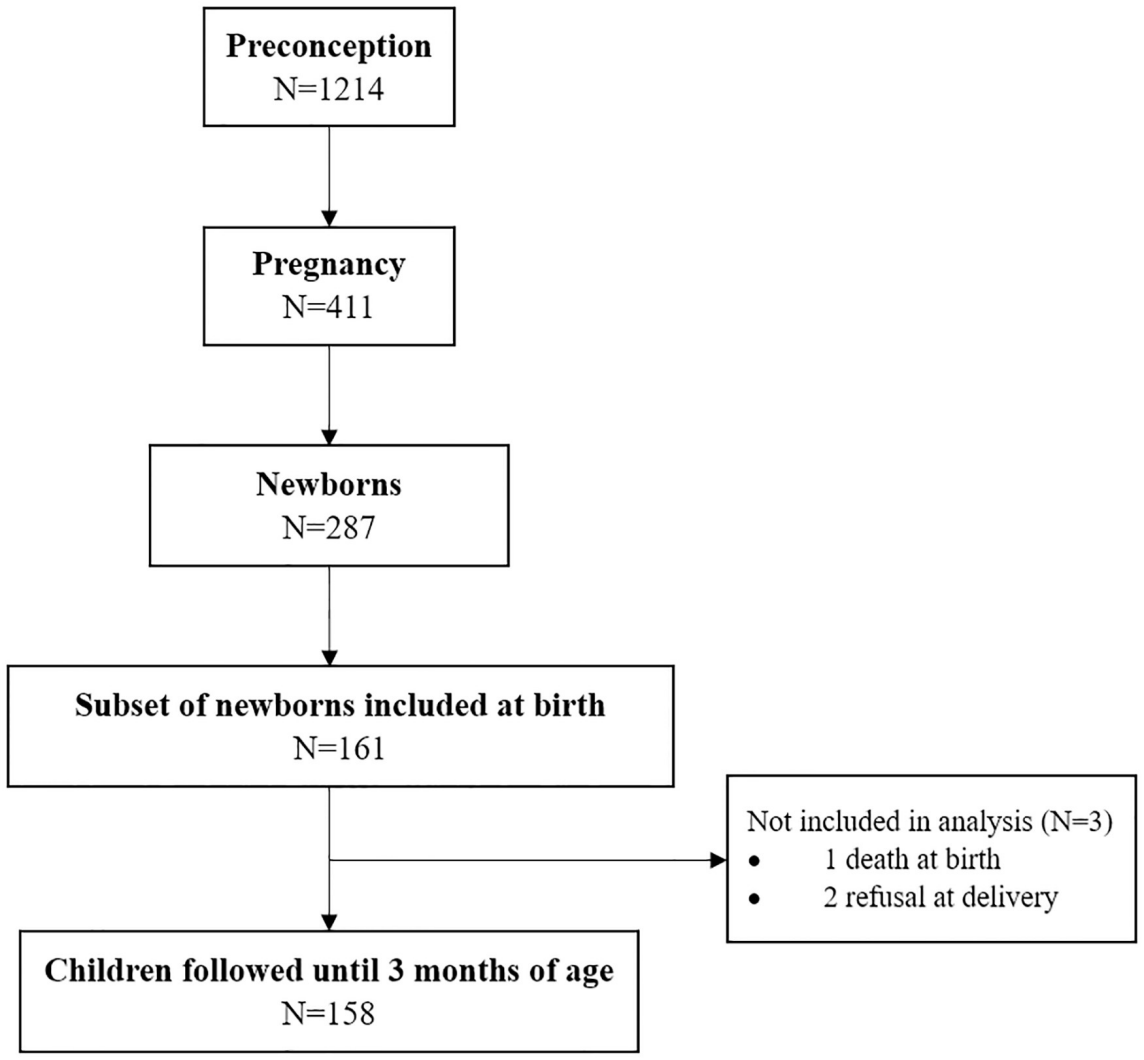
Study profile, Southern Benin, 2014–2018.

Out of a total of 1214 women of reproductive age enrolled in the RECIPAL study, 411 became pregnant. Among them, 273 were followed up until delivery and gave birth to 287 newborns (including 260 singletons, 12 sets of twins and 1 set of triplet). A subset of 161 newborns born from April 2016 was followed up from birth to 3 months of age as part of the SEPSIS study. Among them, one died on the first day of life, and for two more, their parents withdrew the informed consent just after delivery (Fig 1).

### General characteristics of the population

Table 1 shows the general characteristics of the mothers and children included in this study. Most women were illiterate (68.2%), multigravidae (81.5%) and aged 21–30 years old (66.9%). Before conception, the prevalence of malaria was 4.6%. During pregnancy, 35.7% of the women had at least one episode of malaria and among them, 10.8% had early-MiP. The prevalence of maternal urinary schistosomiasis was 21.8% and most of the infections were detected at delivery. According to the study center, the prevalence of maternal urinary schistosomiasis was 40.9% and 2.7% in Sô-Ava and Akassato districts, respectively. The prevalence of malaria-schistosomiasis coinfection was 11.9%.

**Table 1:** Mother-child pairs general characteristics, Southern Benin, 2014–2018.

Characteristics	Categories	Effective	Mean ± SD or %
***Maternal characteristics (n = 157)***			
Age (y)	*All participants*	157	27.0 ± 4.9
	*18–20 y*	20	12.7
	*21–30 y*	105	66.9
	*31–40 y*	32	20.4
Educational level	*Illiterate*	107	68.2
Socioeconomic level	*Low*	40	25.4
	*Medium*	92	58.6
	*High*	25	15.9
Pre-pregnancy BMI*	*Underweight*	16	10.2
	*Normal*	105	66.9
	*Overweight*	27	17.2
	*Obese*	9	5.7
Gravidity	*1–2*	29	18.5
	*≥ 3*	128	81.5
Pre-pregnancy malaria	*Yes*	7	4.6
Malaria in pregnancy (MiP)	*≥ 1 episode(s) during pregnancy*	56	35.7
Timing of MiP	*Early-MiP*	17	10.8
	*Late-MiP*	32	20.4
	*Combined-MiP*	7	4.5
IPTp	*≥ 2 doses*	143	91.1
Possession of ITN	*Yes*	151	96.2
Anemia in pregnancy^‡^	*Yes*	104	66.2
Placental malaria	*Yes*	6	4.1
Urinary schistosomiasis^¥^	*Yes*	32	21.8
Malaria-schistosomiasis coinfection^¥^	*Yes*	18	11.9
***Infant’s characteristics (n = 158)***			
Sex	*Female*	81	51.3
Term at birth (weeks)	*All participants*	158	39.5 ± 1.6
	*Preterm birth (<37 weeks)*	8	5.1
Birth weight (g)	*All participants*	158	3006 ± 376
	*Low birth weight (<2500 g)*	14	8.9
	SGA**	31	20.7
Feeding mode (0-3mo)	*Exclusive breastfeeding*	84	53.2
	*Predominant breastfeeding*^†^	61	38.6
	*Mixed feeding*	13	8.2
	*Exclusive formula feeding*	0	0
Weight-for-length zscore	*< -2 SD at 3mo*	9	5.7
Length-for-age zscore	*< -2 SD at 3mo*	18	11.4
Hb level (g/dL)	*At birth*	158	14.7 ± 2.8
	*At 3 months*	158	10.7 ± 1.5
Febrile infection (0-3mo)^†^	*At least one episode*	40	25.3

Early-MiP: only in the 1^st^ trimester; Late-MiP: In the 2^nd^ and/or 3^rd^ trimester and not in 1^st^; Combined-MiP: 1^st^ trimester combined with 2^nd^ and/or 3^rd^ trimester infection;

^¥^: detected either before or during pregnancy;

^‡^: at least one episode in the 1^st^ or the 3^rd^ trimester;

^†^: water or water-based drinks consumed in addition to breast milk;

*: Body mass index (BMI) in class according to WHO standards;

**: SGA defined according to INTERGROWTH 21^st^ charts;

mo: months, SD: standard deviation; y: years; %: percentage; ITN: insecticide-treated net; IPTp: intermittent preventive treatment in pregnancy with sulfadoxine-pyrimethamine; Missing data: 12 for placental malaria and 10 for maternal schistosomiasis.

Mean (SD) gestational age and weight at birth was 39.5 (1.6) weeks and 3006 (376) grams, respectively. During the first 3 months of life, most infants were exclusively or predominantly breastfed (92%). Mean infant’s Hb concentration at birth and at 3 months of age was 14.7 (2.8) and 10.7 (1.5) g/dL, respectively. During the 3 month-follow-up, 25.3% (40/158) of infants had at least one febrile infection. Respiratory, gastrointestinal and cutaneous infections as well as clinical malaria accounted for 53% (n = 21), 48% (n = 19), 5% (n = 2) and 5% (n = 2) of all cases, respectively.

### Effect of maternal infections on infant’s risk of febrile infection during the first 3 months of life

**Fig 2:**
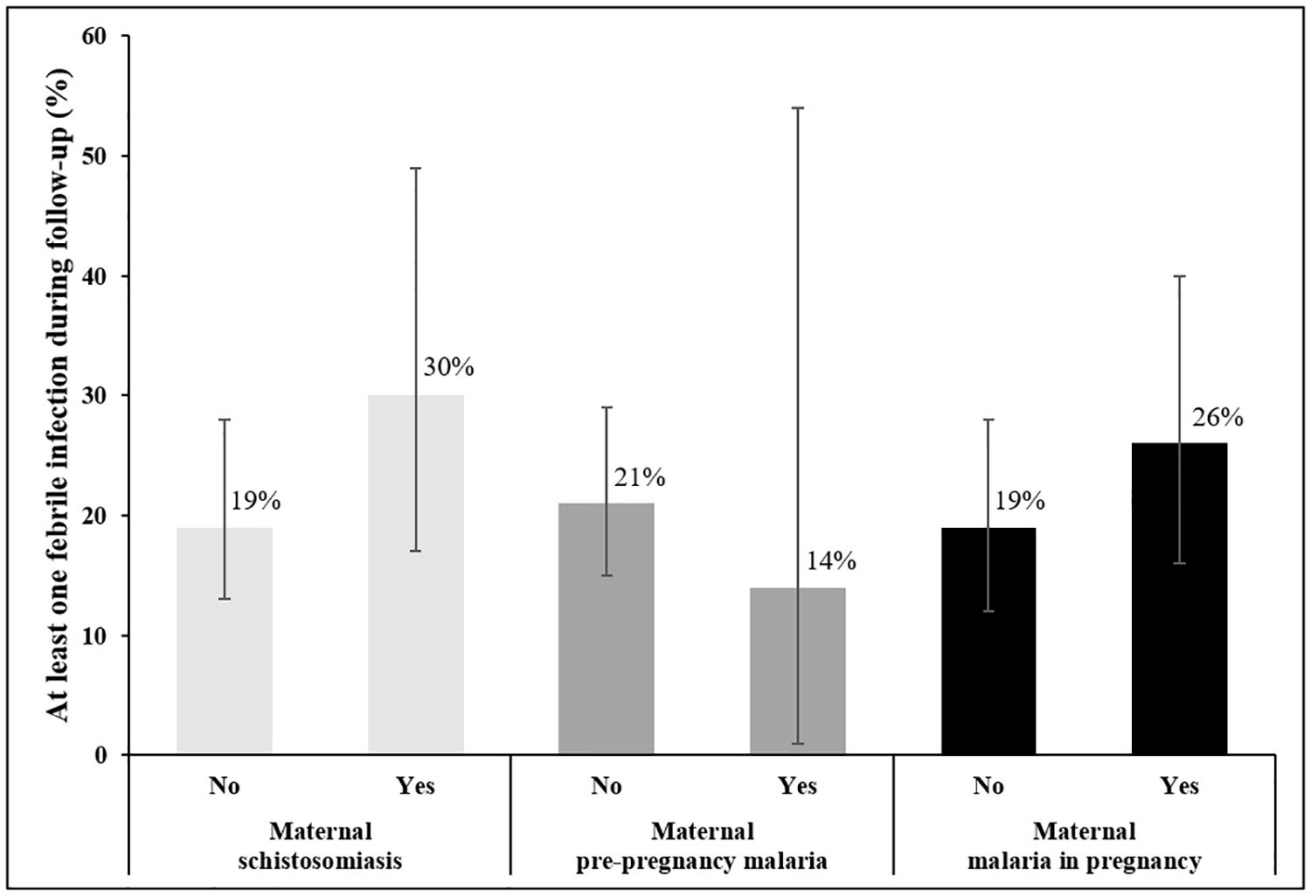
Proportion of infants with at least one febrile infection during the three month-follow-up, according to maternal infection before and during pregnancy, Southern Benin, 2014–2018. (95% confidence interval).

During the first 3 months of life, the proportion of infants with at least one febrile infection varied according to the infected or uninfected status of mother (Fig 2). Indeed, the proportion of febrile infections in infants was high when the mothers had had MiP or schistosomiasis before or during pregnancy and low when the mothers had had malaria before conception compared to non-infected mothers. In multivariate analysis, MiP was significantly associated with the infant’s risk of febrile infection (Adjusted Odds ratio “aOR” = 3.11, 95 CI% [1.11; 8.77], p = 0.032) (Table 2). When considering the timing of MiP, we showed that early-MiP was significantly associated with a higher risk of febrile infection in infants (aOR = 4.99, 95 CI% [1.10; 22.64], p = 0.034). Late-MiP and combined MiP were not significantly associated with infant’s risk of febrile infection. We did not find any association between schistosomiasis or pre-pregnancy malaria and the risk of febrile infection (aOR = 1.40, 95 CI% [0.50; 3.96], p = 0.525 and aOR = 0.50, 95 CI% [0.04; 6.67], p = 0.603, respectively). Similarly, malaria-schistosomiasis coinfection was not significantly associated with the risk of febrile infection. The other factors associated with the infant’s risk of febrile infection are presented in Supplementary S1 Table.

**Table 2:** Association between maternal schistosomiasis and malaria before and during pregnancy and infant’s risk of febrile infection during the first 3 months of life. Logistic regression analyses, n = 149, Southern Benin, 2014–2018.

		Risk of febrile infection			
Variables	Categories	Univariate analysis		Multivariate analysis	
		Unadjusted OR [95% CI]	p-value	Adjusted OR [95% CI]	p-value
***Model 1***					
Pre-pregnancy malaria	*Yes vs*. *No*	0.61 [0.07; 5.28]	0.656	0.50 [0.04; 6.67]	0.603
Maternal schistosomiasis^¥^	*Yes vs*. *No*	1.80 [0.75; 4.32]	0.189	1.40 [0.50; 3.96]	0.525
Malaria during pregnancy*	*Yes vs*. *No*	1.50 [0.70; 3.26]	0.300	3.11 [1.11; 8.77]	0.032
***Model 2 considering the timing of malaria during pregnancy***					
Pre-pregnancy malaria	*Yes vs*. *No*	0.61 [0.07; 5.28]	0.656	0.53 [0.04; 7.55]	0.639
Maternal schistosomiasis	*Yes vs*. *No*	1.80 [0.75; 4.32]	0.189	1.22 [0.39; 3.83]	0.729
Malaria during pregnancy (MiP)	*Early-MiP vs*. *No infection*	1.71 [0.64; 5.44]	0.263	4.99 [1.10; 22.64]	0.034
	*Late-MiP vs*. *No infection*	1.61 [0.64; 4.03]	0.312	2.80 [0.85; 9.23]	0.090
	*Combined-MiP vs*. *No infection*	0.68 [0.08; 6.03]	0.732	1.41 [0.12; 16.43]	0.783
***Model 3 considering malaria-schistosomiasis coinfection***					
Malaria-schistosomiasis coinfection	*Yes vs*. *No*	1.44 [0.47; 4.39]	0.523	2.31 [0.68; 7.89]	0.180

Final model was adjusted for pre-pregnancy AGP level, infant’s nutritional status, breastfeeding, term at birth, low birthweight and study center. CI: confidence interval;

^¥^: detected either before or during pregnancy;

*: at least one microscopic infection during pregnancy

### Secondary results: Effect of maternal infections on infant’s Hb concentration during the first 3 months of life

Overall, Hb concentration tended to decrease over time, with a plateau from 2 months (S1 Fig). The decrease was stronger in infants born from infected mothers (more pronounced for pre-pregnancy malaria and schistosomiasis), but this difference became smaller over time. In multivariate analysis (Supplementary S2 Table), pre-pregnancy malaria and maternal schistosomiasis were significantly associated with a lower infant’s mean Hb concentration during the first 3 months of life (-1.30 g/dL, 95 CI% [-2.27; 0.33], p = 0.009 and -0.63 g/dL, 95 CI% [-1.10; -0.16], p = 0.009, respectively). We did not evidence any association between MiP or malaria-schistosomiasis coinfection and infant’s Hb concentration.

## Discussion

To our knowledge, this is the first study conducted in SSA, which assesses the effect of maternal schistosomiasis and early-MiP on infant’s risk of infection. We evidenced that early-MiP–infection occurring only in the first trimester and not after–was significantly associated with a higher risk of infection in infant during the first 3 months of life.

Thanks to the women’s follow-up from the preconceptional period, we were able to assess the effect of malaria before and in early pregnancy on infant’s infection. The outcomes were collected prospectively and repeatedly from birth to 3 months of age. In addition, important determinants of infant’s infection, such as his nutritional status and feeding practices have been taken into account in the analysis.

In our study, MiP, especially in the 1^st^ trimester of pregnancy was associated with a higher risk of febrile infection. This result is concordant with the literature. Indeed, the association between MiP, particularly placental malaria, and a higher risk of infant’s malaria and non-malaria fever during the first 18 months of life has already been reported [8,10]. These observations have been linked to an immune tolerance phenomenon due to *in utero* exposure to soluble *Plasmodium* antigens. Fetal exposure to malaria may induce an alteration of immune responses leading to a higher susceptibility of infants to subsequent infections, not only to malaria but to infections in general [20–22]. In these studies, only the effect of malaria at delivery or malaria in the third trimester of pregnancy was assessed. In our study, we showed a strong association between women infected in the 1^st^ trimester of pregnancy and subsequent infections in the child, with a 4 times higher risk of infection in infants born from mothers infected in the 1^st^ trimester only. It has been suggested that early malaria in pregnancy may impair placentation and then contribute to the pathogenesis of low birthweight (LBW) and fetal growth restriction, which are high risk factors for morbidity in the child [23]. In our study, the effect of early-MiP was found independently of LBW, suggesting additional underlying mechanisms. We did not evidence an effect of malaria occurring later in pregnancy or a cumulative effect of malaria during pregnancy on the risk of infection in the infant. These results may be explained by the fact that women were treated immediately and repeatedly for malaria during pregnancy and that intermittent preventive treatment coverage (more than 2 doses) was particularly high (91%).

The prevalence of maternal urinary schistosomiasis was high (21.8%), particularly in Sô-Ava district (41%). This result is in line with prevalences that were reported during a recent mapping of *Schistosoma haematobium* infection in schoolchildren in Benin, which were 17.6% at national level and 59.6% in Sô-Ava district [24]. The proportion of infants with a febrile infection tended to be higher in those born from mothers with schistosomiasis than in those born from uninfected mothers, but this association did not reach significance. The higher susceptibility to infection of infants born from mothers chronically infected with helminths found in the literature may be related to changes in fetal and infant’s immune responses such as in utero sensitization of T and B lymphocytes to helminth antigens, leading an immune tolerance (immunosuppressed status) [25]. In our study, the lack of association between maternal urinary schistosomiasis and infant’s infections may be due to the small sample size and number of events. Also, we cannot exclude that women were infected with schistosomiasis late in pregnancy that may not have resulted in changes in fetal immune responses [26].

Looking at the effects on Hb concentrations, we showed that maternal schistosomiasis and pre-pregnancy malaria were significantly associated with a lower infant’s Hb concentration during the first 3 months of life. Maternal schistosomiasis has been related to maternal anemia and LBW, but in a very limited number of studies [27–29]. Anemia due to chronic diseases is one of the proposed mechanisms of schistosomiasis-mediated adverse birth and neonatal outcomes [12]. In particular, schistosomiasis in pregnancy is responsible for iron loss in stool and urine, resulting in maternal iron deficiency and anemia, which has been associated with infant’s hemoglobin concentration in the first months of life [30–32]. However, in our study the effect of schistosomiasis on infant’s Hb concentration was shown after adjustment for maternal anemia, suggesting other underlying mechanisms. Malaria at delivery has already been associated with a lower Hb concentration in infants during the first year of life [33]. Here we found an association between pre-pregnancy malaria and infant’s Hb concentration. This association remained statistically significant after controlling for potential confounders such as malaria during pregnancy or other maternal characteristics that may be linked to poor conditions in the child. This result relies on Hb concentrations that were measured from birth to 3 months of age, a period during which Hb varies greatly physiologically. Further studies are needed to confirm this over a longer period of time.

Our study has some limitations that should be considered. First, urinary schistosomiasis was detected before pregnancy and all infected women were treated immediately. It is likely that schistosomiasis detected at the end of pregnancy may have occurred later in women, resulting in a limited effect on the fetus. Second, because of our small sample size, we could not assess the effect of schistosomiasis, malaria and malaria-schistosomiasis coinfection according to the intensity of these infections.

In conclusion, our results highlight the critical effects of maternal infections during the first 1,000 days of life in line with the DOHaD concept. We showed that maternal schistosomiasis and malaria in the peri-conceptional period were independently associated with a higher risk of infant’s febrile infection and Hb concentration during the first 3 months of life. These results underline the need for measures to prevent maternal infections as early as in the peri-conceptional period.

## Supporting information

S1 Fig Evolution of infant’s hemoglobin (Hb) concentration during the first 3 months of life according to maternal schistosomiasis and malaria before and during pregnancy, Southern Benin, 2014–2018. Schisto: schistosomiasis.
(TIF)

S1 Table Association between maternal schistosomiasis and malaria before and during pregnancy and infant’s risk of febrile infection during the first 3 months of life, uni and multivariate logistic regression analyses, n = 140, Southern Benin, 2014–2018. Malaria and schistosomiasis have been forced in all final models; Breast: breastfeeding included exclusive and predominant feeding [18]. Infant’s weight-for-length, weight-for-age and length-for-age z-scores are time dependent variable.
(DOCX)

S2 Table Relationship between infant’s hemoglobin concentration during the first 3 months of life and maternal urinary schistosomiasis and malaria before and during pregnancy, uni and multivariate mixed linear regression analyses, n = 148, Southern Benin, 2014–2018. Malaria and schistosomiasis have been forced in all final models; Breast.: breastfeeding included exclusive and predominant feeding [18]. Infant’s weight-for-length, weight-for-age and length-for-age z-scores are time dependent variable.
(DOCX)
