# Supplementary figures and images for: Maternal malaria but not schistosomiasis is associated with a higher risk of febrile infection in infant during the first 3 months of life: A mother-child cohort in Benin
Source: PLoS One. 2019 Sep 19;14(9):e0222864. doi: 10.1371/journal.pone.0222864 (PMC6752763; doi:10.1371/journal.pone.0222864)

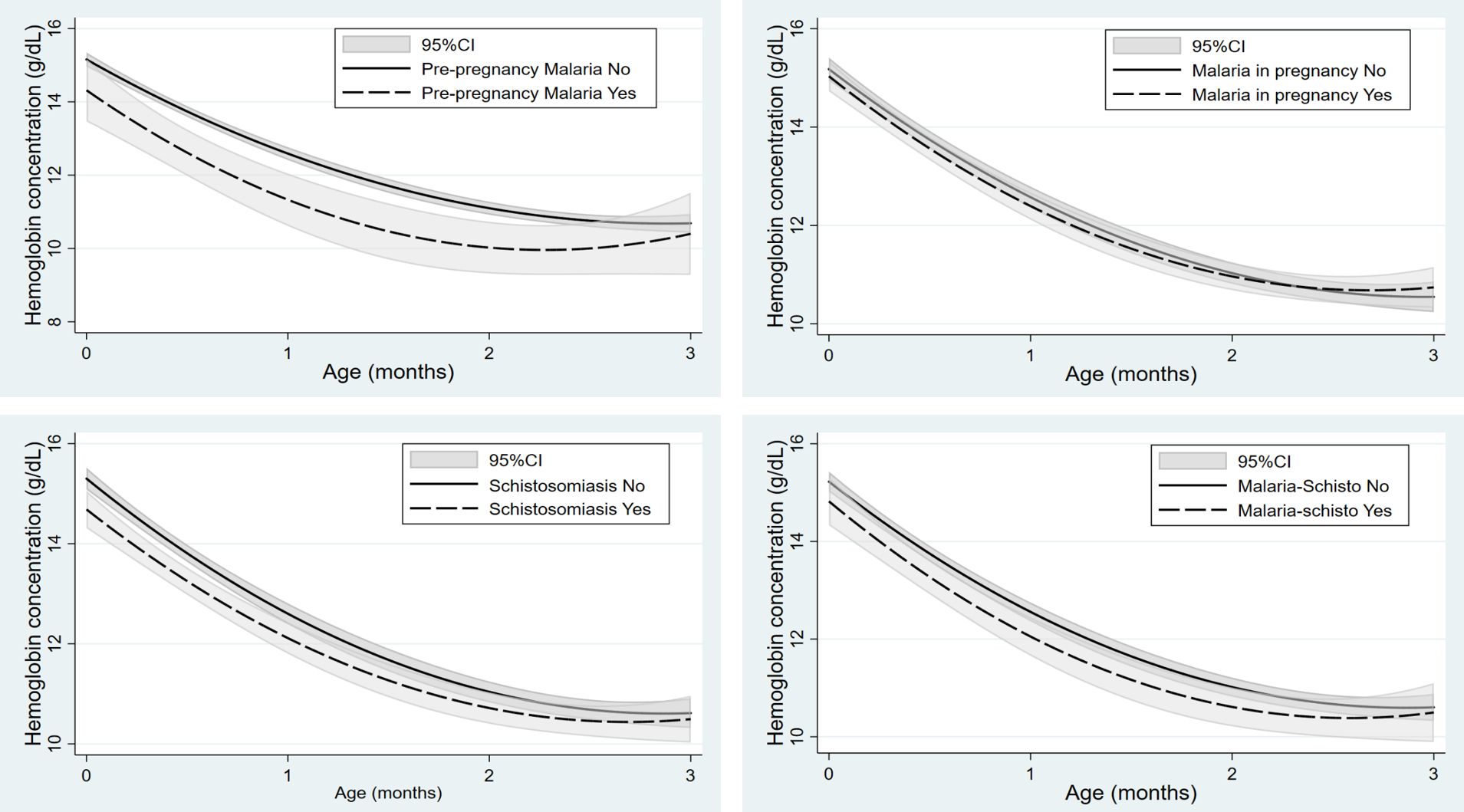

Supplement: S1 Fig — Schisto: schistosomiasis. (TIF) [file pone.0222864.s001.tif]
