# Supplementary material for: Maternal malaria but not schistosomiasis is associated with a higher risk of febrile infection in infant during the first 3 months of life: A mother-child cohort in Benin
Source: PLoS One. 2019 Sep 19;14(9):e0222864. doi: 10.1371/journal.pone.0222864 (PMC6752763; doi:10.1371/journal.pone.0222864)
Supplement: S1 Table — Malaria and schistosomiasis have been forced in all final models; Breast: breastfeeding included exclusive and predominant feeding [18]. Infant’s weight-for-length, weight-for-age and length-for-age z-scores are time dependent variable. (DOCX) [file pone.0222864.s002.docx]

S1 Table. Association between maternal schistosomiasis and malaria before and during pregnancy and infant’s risk of febrile infection during the first 3 months of life, uni and multivariate logistic regression analyses, n=140, Southern Benin, 2014-2018

| Variables | Categories | Univariate analysis | |  | Multivariate analysis | |
| --- | --- | --- | --- | --- | --- | --- |
|  |  | Unadjusted OR [95% CI] | p-value |  | Adjusted OR [95% CI] | p-value |
| *Model 1* |  |  |  |  |  |  |
| Pre-pregnancy malaria | *Yes vs. No* | 0.61 [0.07; 5.28] | 0.656 |  | 0.50 [0.04; 6.67] | 0.603 |
| Maternal schistosomiasis | *Yes vs. No* | 1.80 [0.75; 4.32] | 0.189 |  | 1.40 [0.50; 3.96] | 0.525 |
| Malaria during pregnancy | *Yes vs. No* | 1.50 [0.70; 3.26] | 0.300 |  | 3.11 [1.11; 8.77] | 0.032 |
| Pre-pregnancy high level of AGP (≥1 g/L) | *Yes vs. No* | 3.96 [1.31; 11.92] | 0.015 |  | 5.08 [1.07; 24.04] | 0.040 |
| Anemia during pregnancy | *Yes vs. No* | 0.99 [0.45; 2.21] | 0.996 |  |  |  |
| Maternal educational status | *Illiterate vs. literate* | 2.10 [0.84; 5.22] | 0.110 |  |  |  |
| Infant’s weight-for-length z-score at 3 months |  | 0.91 [0.68; 1.22] | 0.547 |  | 1.01 [0.54; 1.87] | 0.980 |
| Preterm birth (< 37 weeks) | *Yes vs. No* | 3.93 [0.93; 16.65] | 0.063 |  | 8.85 [1.00; 78.04] | 0.050 |
| Low birth-weight (< 2500 g) | *Yes vs. No* | 3.05 [0.98; 9.51] | 0.054 |  | 7.13 [1.45; 35.02] | 0.016 |
| Infant’s sex | *Female vs. Male* | 1.35 [0.63; 2.90] | 0.439 |  |  |  |
| Feeding mode (0-3 months) | *Mixt vs. Breast.* | 1.67 [0.48; 5.81] | 0.417 |  | 0.48 [0.06; 3.58] | 0.475 |
| Infant’s Hb level at birth |  | 1.01 [0.87; 1.18] | 0.864 |  |  |  |
| Infant’s Hb level at 3 months |  | 0.90 [0.67; 1.20] | 0.478 |  |  |  |
| Study center | *Akassato vs .Sô-Ava* | 0.66 [0.30; 1.46] | 0.310 |  | 0.67 [0.22; 2.05] | 0.484 |

Malaria and schistosomiasis have been forced in all final models; Breast: breastfeeding included exclusive and predominant feeding [18]. Infant’s weight-for-length, weight-for-age and length-for-age z-scores are time dependent variable
