# Supplementary material for: Maternal malaria but not schistosomiasis is associated with a higher risk of febrile infection in infant during the first 3 months of life: A mother-child cohort in Benin
Source: PLoS One. 2019 Sep 19;14(9):e0222864. doi: 10.1371/journal.pone.0222864 (PMC6752763; doi:10.1371/journal.pone.0222864)
Supplement: S2 Table — Malaria and schistosomiasis have been forced in all final models; Breast.: breastfeeding included exclusive and predominant feeding [18]. Infant’s weight-for-length, weight-for-age and length-for-age z-scores are time dependent variable. (DOCX) [file pone.0222864.s003.docx]

S2 Table. Relationship between infant’s hemoglobin concentration during the first 3 months of life and maternal urinary schistosomiasis and malaria before and during pregnancy, uni and multivariate mixed linear regression analyses, n=148, Southern Benin, 2014-2018

| Variables | Categories | | Univariate analysis | |  | Multivariate analysis | |
| --- | --- | --- | --- | --- | --- | --- | --- |
|  |  |  | Unadjusted β [95% CI] | p-value |  | Adjusted β [95% CI] | p-value |
| Pre-pregnancy malaria | | *Yes vs. No* | -1.06 g/dL [-2.01; -0.11] | 0.028 |  | -1.30 g/dL [-2.27; -0.33] | 0.009 |
| Maternal schistosomiasis | | *Yes vs. No* | -0.34 g/dL [-0.84; 0.16] | 0.182 |  | -0.63 g/dL [-1.10; -0.16] | 0.009 |
| Malaria during pregnancy | | *Yes vs. No* | -0.05 g/dL [-0.46; 0.36] | 0.815 |  | 0.17 g/dL [-0.23; 0.57] | 0.407 |
| Pre-pregnancy high level of AGP (≥ 1 g/L) | | *Yes vs. No* | -0.14 g/dL [-0.82; 0.53] | 0.677 |  |  |  |
| Pre-pregnancy anemia (< 12 g/dL) | | *Yes vs. No* | -0.09 g/dL [-0.48; 0.31] | 0.675 |  |  |  |
| Anemia during pregnancy | | *Yes vs. No* | -0.14 g/dL [-0.56; 0.18] | 0.189 |  | -0.40 g/dL [-0.84; 0.03] | 0.067 |
| Infant’s weight-for-length z-score | |  | -0.44 g/dL [-0.59; -0.28] | <0.001 |  | -0.47 g/dL [-0.63; -0.31] | <0.001 |
| Infant’s weight-for-age z-score | |  | 0.00 g/dL [-0.19; 0.19] | 0.988 |  |  |  |
| Infant’s length-for-age z-score | |  | 0.26 g/dL [0.09; 0.43] | 0.003 |  |  |  |
| Sex | | *Female vs. Male* | 0.13 g/dL [-0.27; 0.52] | 0.537 |  | 0.13 g/dL [-0.26; 0.52] | 0.515 |
| Feeding mode (0-3 months) | | *Mixt vs Breast.* | -0.70 g/dL [-1.42; 0.03] | 0.060 |  | -0.38 g/dL [-1.15; 0.40] | 0.342 |
| Preterm birth (< 37 weeks) | | *Yes vs. No* | 0.35 g/dL [-0.60; 1.30] | 0.471 |  | 0.08 g/dL [-0.92; 1.08] | 0.874 |

Malaria and schistosomiasis have been forced in all final models; Breast.: breastfeeding included exclusive and predominant feeding [18]. Infant’s weight-for-length, weight-for-age and length-for-age z-scores are time dependent variable
